# The Homeless People in China During the COVID-19 Pandemic: Victims of the Strict Pandemic Control Measures of the Government

**DOI:** 10.3389/fpubh.2021.679429

**Published:** 2021-08-12

**Authors:** Ying Wang, Lei Hua, Shuyun Zou, Taofeng Deng, Yongqi Chen, Wanying Cao, Chuhan Wu, Yujie Zhou, Hua Zou

**Affiliations:** ^1^School of Public Policy and Administration, Chongqing University, Chongqing, China; ^2^School of Public Administration, Nanfang College of Sun Yat-sen University, Guangzhou, China; ^3^College of Attainment, Nanfang College of Sun Yat-sen University, Guangzhou, China; ^4^School of Government, Sun Yat-sen University, Guangzhou, China

**Keywords:** COVID-19 pandemic, homeless people, Mainland China, government's strict pandemic control, humanitarian crisis

## Abstract

**Background:** By implementing aggressive control measures, China has rapidly and effectively controlled the Coronavirus disease 2019 (COVID-19) pandemic. However, the neglected homeless population may become victims of that perceived success. Due to political sensitivity, we know little about them.

**Aims:** This study aimed to investigate how the pandemic and the pandemic control strategies of the government affected the lives of the homeless people in mainland China.

**Methods:** A total of 103 eligible participants experiencing absolute homelessness were recruited from Guangzhou City during July and August 2020. Surveys measured demographic characteristics including health status, changes in daily living, and actions of the government toward the homeless during the pandemic. Sankey diagrams and ordered logit regression models were used to examine the impact on the homeless of inhumane government efforts to drive the homeless away. Qualitative materials were analyzed by using an inductive approach to provide more details.

**Results:** First, the homeless people in Guangzhou tended to be male, aged 40 to 64 years, less well-educated, and they originated from outside Guangdong although they were living in the city center. Most had little connection with their families. After a long period of homelessness, almost half of the participants were in poor health with various conditions, which made them extremely vulnerable to COVID-19. Second, the pandemic caused a substantial decline in incomes of homeless people, had less of a negative impact on their food intake, and affected sleep time in different ways. Third, during the COVID-19 pandemic, humanitarian aid from local governments of China decreased, whereas inhumane efforts to drive the homeless away intensified. Fourth, quantitative models and qualitative materials demonstrate the devastating effect of the strict pandemic control strategy of the government on the lives of homeless people, which may further cause their health problems.

**Conclusion:** This study for the first time illustrated the characteristic features of the street homeless population in mainland China and their living situation during the COVID-19 pandemic, and most importantly demonstrated the devastating effect of the strict pandemic control of the government, which has been considered a great success in previous studies, on lives of homeless people. Urgent measures should be taken to ensure the protection of the homeless population and prevent an impending humanitarian crisis.

## Introduction

Coronavirus disease 2019 (COVID-19), which was caused by the novel coronavirus SARS-CoV-2, was first identified in Wuhan City, Hubei Province, China, in late December 2019, and has now spread rapidly around the world. According to the WHO Coronavirus Disease Situation Dashboard, there had been more than 133.14 million confirmed cases globally and more than 2.88 million deaths by April 12, 2021. The numbers continue to increase rapidly as we are writing. During these challenging times, academia played a crucial role in combating COVID-19 and has published many articles related to the pandemic, and the number of published articles is rapidly accumulating. However, the existing literature pays inadequate attention to the homeless, who are considered the most vulnerable group to COVID-19 (1–5). The limited literature on the homeless focuses more on introducing or proposing various strategic measures and policy implications to support the homeless population during the COVID-19 epidemic (6–16), describing the characteristics and outbreak of COVID-19 in homeless shelters (17–20), or addressing the mental health status of the homeless during the pandemic (21–23). There are hardly any exploratory efforts and no evidence to show the impact of the COVID-19 pandemic and the pandemic control strategies of the government on the lives of the homeless in China. The economic and social disruption caused by the COVID-19 pandemic brings an unprecedented challenge to the lives of everyone. However, the homeless are more vulnerable and have been particularly impacted by the pandemic and the actions of the government (24), such as extremely unstable living situations and the lack of a living wage and savings. This group, therefore, requires additional support.

In mainland China, due to the imperfect relief policy for the homeless, they are often treated unfairly and carelessly. On the other hand, public opinion has gradually shown more and more attention to non-humanitarian experiences, which can easily lead to scandals that may damage the positive image of the party and the government (25–29). In this context, the homeless have become a highly politically sensitive issue. As a result, public discussions and academic research on disadvantaged groups have been suppressed, and the homeless have been ignored. There is minimal literature on the homeless in China, and the existing studies focus mainly on mental illness (30–33). To the best of our knowledge, there is no survey-based study that attempts to examine the impact of the COVID-19 pandemic, particularly the impact of the strict pandemic control measures of the government on the lives of the homeless in China.

Although the COVID-19 pandemic has been traumatic, if the government implements strict pandemic control measures which prefer inhumane actions rather than humanitarian aid, it may cause devastating damage to the lives of the homeless. In mainland China, the government manages the homeless in cities in three ways: sending them back to their hometowns, providing them with temporary shelters, and driving them off. The first two actions seem humane because, under current regulations, the homeless own the right to accept or refuse assistance, and government staff can only provide advice rather than influence the homeless against their will (34). If the homeless give their approval, they receive free food, accommodation, and train tickets to their hometowns (34). However, during the COVID-19 pandemic, the government implemented strict lockdown measures to prevent the spread of coronavirus by closing off all villages and residential compounds nationwide. Thus, local governments stopped sending the homeless back to their hometowns. Meanwhile, the shelters were at high risk and under enormous pressure because the coronavirus could be rapidly and widely transmitted in homeless shelters (18, 20, 35, 36). In addition, the shelters had also been ordered to isolate all new entrants, which was another difficult task. Compared to the situation before the pandemic, shelters faced overwhelming demand that extended far beyond the need for bedding (37). As a result, local shelters lacked the will and the ability to help the homeless during the pandemic.

The humanitarian aid provided by the government was reduced while efforts to drive the homeless away intensified. The intensified eviction efforts were the most striking feature of the pandemic control strategy of the Chinese government for the homeless and had been adopted by governments at all levels across the country during the pandemic (38–41). Under the guidance of aggressive pandemic prevention and control, local governments expelled the homeless to prevent cross-infection caused by the integration of the homeless in densely populated city centers. Meanwhile, eviction efforts of the government also prevented the homeless, who live by collecting garbage, from being infected by household garbage of unknown origin. In addition, under the pressure of the “hold-to-account” system, which imposes immediate and harsh sanctions for negative results of local government pandemic control (42), local officials feared being punished or fired for the increasing number of COVID-19 cases. Since the homeless was one of the most vulnerable groups to COVID-19, and few people care about them, driving the homeless away from jurisdictions became the best option for local governments.

Therefore, as a result of strict pandemic control measures, many of the homeless in China may not be able to return to their hometowns or find shelter. Critically, during the pandemic, the government may continue to expel the homeless. Scholars have credited the success of China in controlling COVID-19 to aggressive control measures (43), but the perceived success may conceal the huge sacrifice of the neglected homeless. Although the Chinese government claimed that they took the initiative during the pandemic and successfully helped the homeless by searching the streets each day. We doubt whether they had sufficient government personnel to do this as they were already facing serious staff shortages before the pandemic (34, 37, 44).

With little aid but intensified eviction efforts, the living conditions of the homeless people may be worse. Thus, we hypothesized that the strict pandemic control strategy of the government toward the homeless, which preferred driving the homeless away rather than providing humanitarian aid, had a devastating impact on the lives of homeless people.

This study aimed to investigate how the pandemic and the pandemic control strategies of the government affected the lives of the homeless people in mainland China. To achieve that goal, the researchers conducted a street-based survey on individuals who were experiencing “absolute homelessness” (sleeping rough with no shelter or accommodation) (45, 46) in Guangzhou City, China. Based on the basic characteristic data of the homeless collected by the survey, the first research objective was to present pertinent characteristics, particularly the health status of the street homeless in China. The second research objective was to compare the incomes (47, 48), food intake (49, 50), and sleep time (51, 52) of the street homeless, before and during the COVID-19 pandemic, to further illustrate the change that occurred in the lives of the homeless. The third objective was to capture the actions of the local government of China toward the homeless before and during the pandemic. The final objective was to investigate the impact of the efforts of the government to drive the homeless away.

## Methods

### Setting and Participants

We conducted a street-based, cross-sectional, mixed-method survey of the homeless in Guangzhou City, Guangdong Province, China, during July and August 2020. We selected Guangzhou, one of the most developed cities in China, as the location for this study given its large homeless population and its superior performance in homeless assistance (53). Individuals experiencing absolute homelessness were eligible to participate in the study. The study was reviewed and approved by School of Public Administration, Nanfang College of Sun Yat-sen University. The participants were informed that their participation was voluntary, and confidentiality was assured. By responding to the survey, they agreed to participate in the research study; therefore, a signed informed consent was not obtained (54, 55).

### Sampling Method

In previous homeless research, participants were recruited from homeless shelters (18, 35, 56–64), which are called rescue stations in China, or public places, such as streets, under bridges, and parks (61, 63, 65–71). Rescue stations of China were established primarily to help the homeless and beggars; however, rescue stations were exposed as places where vulnerable groups have suffered torture and assault over time (25). Since then, rescue stations in China have become inaccessible places shrouded in secrecy and political sensitivity. Thus, the only way we could reach the homeless population was by conducting street surveys.

To find eligible participants, we first contacted local NGOs that attempt to improve the lives of the homeless. We also contacted vegetarian restaurants in Guangzhou that regularly provide hot food for the homeless. In China, Buddhism is closely associated with vegetarianism (72–74). By following the Buddhist ethics that encourage giving and helping others, vegetarian restaurants in China usually cooperate with NGOs to provide assistance for the homeless people (75). From these sources, we obtained a list of locations where the homeless usually gather and sleep. The interviewers then looked for people who appeared to be homeless by following the list of locations. We noticed that almost all street homeless people lived in downtown Guangzhou, including Yuexiu District, Haizhu District, Liwan District, and Tianhe District. Out of the list, we also walked the streets in the outskirts of Guangzhou, including Huangpu District, Panyu District, Baiyun District, Huadu District, Conghua District, Zengcheng District, and Nansha District, to identify potential participants and reduce potential sample selection bias.

### Measures

The homeless people were assessed utilizing quantitative and qualitative measures. The quantitative measures were used to illustrate the characteristic features of the street homeless population in mainland China and their living situation during the COVID-19 pandemic. However, by simply relying on the results drawn from emotionless data, we may not fully appreciate what the homeless suffered during the pandemic. The experiences and feelings the respondents shared with us provide insights into their tragic circumstances. Therefore, to uncover the action of the local government of China toward the homeless and its effect on the lives of the homeless during the pandemic, both quantitative and qualitative measures were used.

#### Quantitative Measures

##### Basic Characteristics

**Figure 1:**
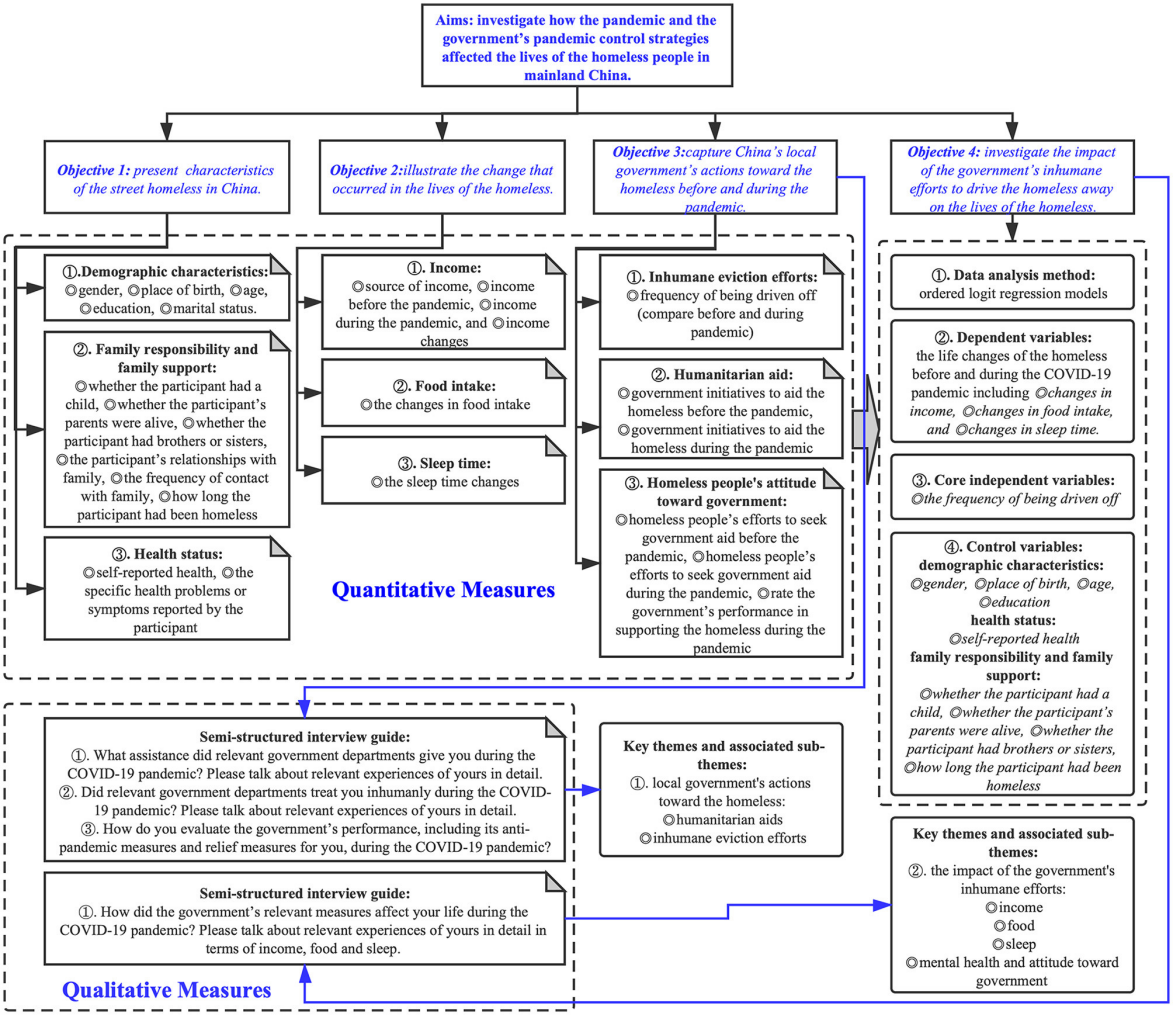
Flowchart of the research process.

By following the previous literature, this survey captured the basic characteristics of respondents in three dimensions: demographic characteristics, family responsibility and family support, and health status (see Figure 1). The first dimension, demographic characteristics, included gender (45, 67, 68, 76, 77), place of birth (31), age (45, 64, 67, 68, 77–79), education (45, 64, 79), and marital status (64, 67, 77, 79). The second dimension, family responsibility, and family support, included whether the participant had a child (24, 79), whether parents of the participant were alive (76), whether the participant had brothers or sisters (80), relationships of the participant with family (81), the frequency of contact with family (82), and how long the participant had been homeless (64, 67, 68). The third dimension, health status, included self-reported health (78, 83) and the specific health problems or physical signs reported by the participant (78). It is worth mentioning that we obtained the information about health problems or physical signs of homeless people by asking them “Have you ever been told by a doctor, nurse, or other health professionals that you have some kind of disease?” (78, 84, 85). Respondents stating “yes” were asked to specify what kind of disease (physical sign) it is (86, 87).

##### The Changes in the Lives of Homeless People Before and During the Pandemic

The changes in the lives of homeless people before and during the COVID-19 pandemic (from January 23, 2020, to March 29, 2020) were our main concern and our dependent variables in the quantitative models. Based on the previous literature, this study measured the lives of homeless people from three aspects that can easily be answered and compared by respondents: income (47, 48), food intake (49, 50), and sleep time (51, 52) (see Figure 1). Income is a key factor affecting the standard of living among the homeless, which was evaluated through the following survey items: source of income (77), income before the pandemic (67), income during the pandemic (67), and income changes (24) (decrease, constant, and increase). By asking the participants to describe the specific types and quantities of food that they typically consume before and during the pandemic, we assessed the changes in food intake (88, 89) (decrease, constant, and increase). In fact, due to low incomes, the homeless have few options in food, so their daily diets are usually monotonous and constant, which can be easily recalled. In addition, by comparing the sleep time reported by participants before and during the pandemic, we assessed the sleep time changes (90) (decrease, constant, and increase).

##### Action of the Government Toward the Homeless Before and During the Pandemic

This study investigated the impact of the strict pandemic control measures of the government on the lives of the homeless. As mentioned in the introduction, under the guidance of the strict pandemic control, humanitarian aid from the government might decrease, whereas inhumane efforts to drive the homeless away might increase. These inhumane eviction efforts were the most striking feature of the pandemic control strategy of the Chinese government toward the homeless. This study focused on the inhumane efforts of the government as our core independent variable by asking participants to compare the frequency with which they were driven off by government staff before and during the COVID-19 pandemic (never, less frequently, as usual, more frequently) (see Figure 1). We also asked participants whether the local government provided them with aid (yes/no, before/during the pandemic) and whether the participants sought aid from the government (yes/no, before/during the pandemic). Finally, we asked participants to rate the performance of the government in supporting the homeless during the pandemic. What is worth mentioning is that, due to the limited literature, the variables in this section were developed through a combination of focus groups and expert consultation.

#### Qualitative Measures

The homeless were interviewed by a semi-structured interview to explore their experiences and opinions of the action of the local government toward them and its effect on their lives. This allowed the development of the emerging themes for further analysis (91) (the interview guide is presented in Figure 1). Due to the limited literature, the interview was also developed through a combination of focus groups and expert consultation.

### Data Collection

**Figure 2:**
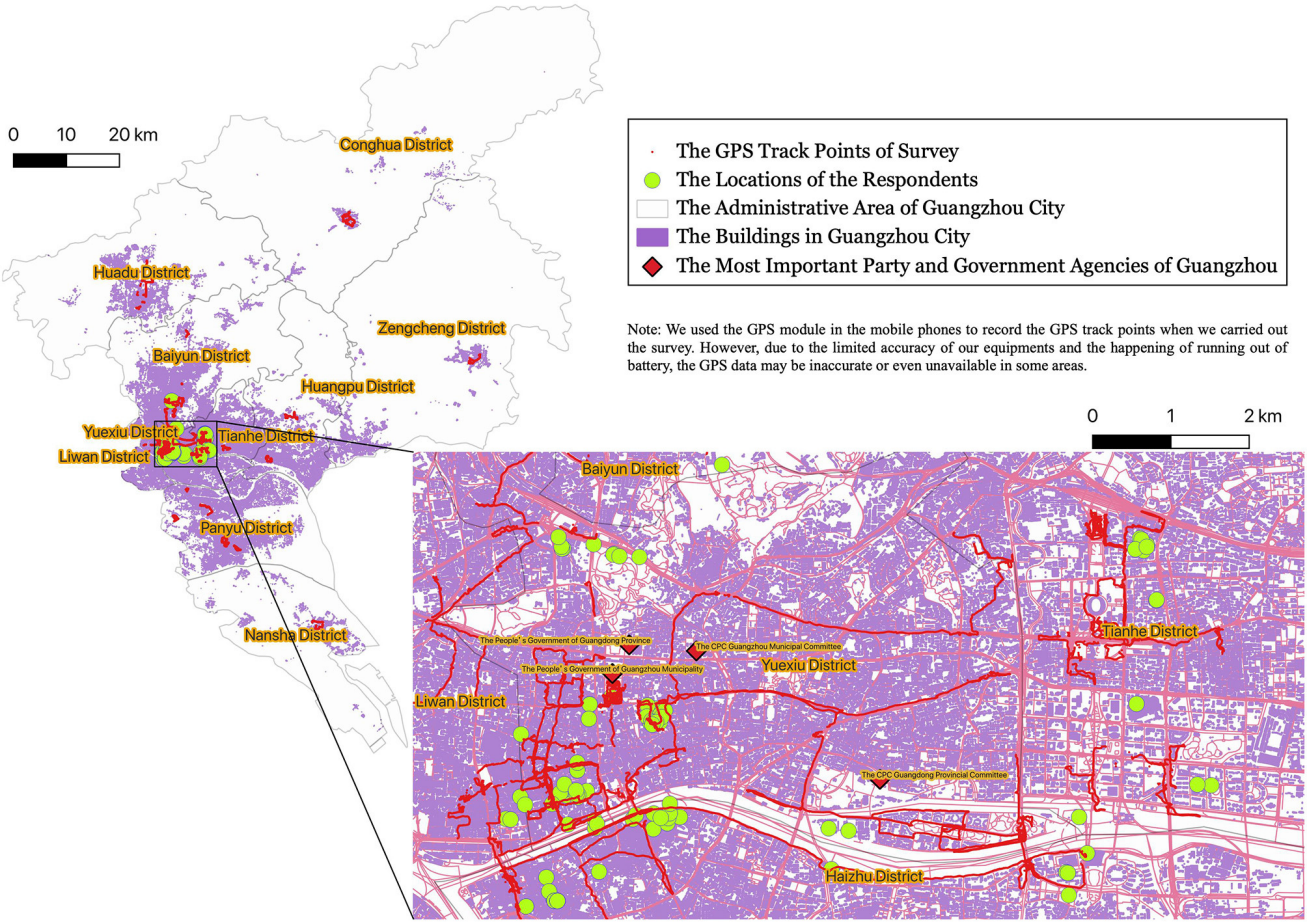
The geographical characteristics of respondents.

Street-based, face-to-face, mixed-method surveys were conducted between July and August 2020 by four research teams. Each team was composed of three well-trained interviewers so that one could focus on the interviewee, while the others could fill in the questionnaire, make on-site records, and ask for a follow-up to answer questions from a different perspective (92). Participants underwent a mixed-method survey, including demographic data, quantitative and qualitative issues related to health status, changes in daily life (income, food intake, and sleep time) during the pandemic, and the actions of the government toward the homeless during the pandemic (61). To protect the privacy of participants and encourage them to share their true thoughts and feelings, the conversation was not audiotaped. After each survey, the interviewer compared the interpretations to avoid any interpretation bias. In addition, we used mobile phone GPS modules to record the locations where we encountered the participants and to mark the tracks on the map as the survey team walked or cycled along the streets (see Figure 2) (93). A total of 103 qualified participants who were experiencing absolute homelessness were recruited from public places, including streets, railway stations, bus stations, temples, under bridges, and parks. On average, each survey took ~21 min to complete.

### Statistical Analysis

#### Quantitative Data Analysis

We used descriptive statistics to report demographic information, details on the lives of homeless people, and the actions of the government toward the homeless (see Tables 1–3). We used the ordered logit regression models to examine the impact of the inhumane efforts of the government to drive the homeless away on their lives. The likelihood-ratio (LR) chi-square with a *p*-value was also reported in each ordered logit regression model to indicate whether the model as a whole was statistically significant or not, as compared to the null model with no predictors.

**Table 1:** Basic characteristics of the respondents (*n* = 103).

**Characteristic**	**Frequency**	**Percentage (%)**
**Gender**		
Female = 0	8	7.77
Male = 1	95	92.23
**Place of birth**		
Guangdong = 1	30	29.13
Henan = 0	11	10.68
Guangxi = 0	10	9.71
Hunan = 0	9	8.74
Gansu = 0	6	5.83
Prefer not to say = None	6	5.83
Shandong = 0	5	4.85
Hubei = 0	4	3.88
Inner Mongolia = 0	4	3.88
Jiangxi = 0	4	3.88
Hebei = 0	3	2.91
Liaoning = 0	3	2.91
Anhui = 0	2	1.94
Sichuan = 0	2	1.94
Chongqing = 0	1	0.97
Jiangsu = 0	1	0.97
Ningxia = 0	1	0.97
Shanghai = 0	1	0.97
**Age**		
25–29 = 1	3	2.91
30–34 = 2	6	5.83
35–39 = 3	7	6.80
40–44 = 4	12	11.65
45–49 = 5	10	9.71
50–54 = 6	14	13.59
55–59 = 7	16	15.53
60–64 = 8	17	16.50
65–69 = 9	7	6.80
70–74 = 10	8	7.77
75–79 = 11	1	0.97
80–84 = 12	2	1.94
**Education**		
College and university undergraduate = 3	2	1.94
Senior high school = 2	21	20.39
Junior high school and below = 1	78	75.73
Prefer not to say = None	2	1.94
**Marital status**		
Married	32	31.07
Divorced	20	19.42
Never married	49	47.57
Prefer not to say	2	1.94
**Whether the participant had a child**		
Yes = 1	44	42.72
No = 0	57	55.34
Prefer not to say = None	2	1.94
**Whether the parents of the participants were alive**		
Alive = 1	32	31.07
Passed away = 0	62	60.19
Prefer not to say = None	9	8.74
**Whether the participant had brothers or sisters**		
Yes = 1	77	74.76
No = 0	24	23.30
Prefer not to say = None	2	1.94
**Relationships with family**		
Good	26	25.24
Fair	28	27.18
Poor	22	21.36
No family	11	10.68
Prefer not to say	16	15.53
**Frequency of contact with family**		
Maintain constant contact	15	14.56
Rare contact	23	22.33
Completely no contact	47	45.63
No family	11	10.68
Prefer not to say	7	6.80
**How long the participant had been homeless**		
<1 year = 1	22	21.36
1–2 years = 2	9	8.74
2–3 years = 3	8	7.77
3–4 years = 4	6	5.83
4–5 years = 5	4	3.88
5–10 years = 6	15	14.56
10–20 years = 7	17	16.50
20–30 years = 8	11	10.68
Over 30 years = 9	10	9.71
Prefer not to say = None	1	0.97
**Self-Reported health**		
Healthy = 0	54	52.43
Unhealthy = 1	49	47.57
**Health problems or physical signs reported by participants (** ***n*** **= 49)**		
Prefer not to say	12	24.49
Cripple	7	14.29
Stroke experience	5	10.20
Heart disease	4	8.16
Hypertension	4	8.16
Lung disease, such as emphysema, asthma, and bronchitis	4	8.16
Eye disease, such as cataracts and cysts in eye	4	8.16
Skin diseases	4	8.16
Diabetes mellitus	2	4.08
Dizziness	2	4.08
Gout	2	4.08
Fracture	2	4.08
Kidney diseases	1	2.04
Lumbar disk herniation	1	2.04
Tumor	1	2.04
Dysaudia	1	2.04
Post-Polio syndrome	1	2.04
Osteoporosis	1	2.04
Headache and nausea	1	2.04
Functional decline of organs	1	2.04
Stomach disease	1	2.04
Malnutrition and low immunity	1	2.04
Diarrhea	1	2.04
Hypotension	1	2.04
Fever	1	2.04
Hand ache	1	2.04
Leg edema	1	2.04
Some sort of chronic disease	1	2.04
Upper respiratory infection	1	2.04

*Of the 49 homeless people who reported being unhealthy, 37 reported their health problems or physical signs, while 12 preferred not to say*.

The life changes of the homeless before and during the COVID-19 pandemic are the dependent variables in this study and are composed of three components: changes in income, changes in food intake, and changes in sleep time. The changes were divided into three ordered categories: decrease, constant, and increase, coded as 1, 2, and 3, respectively. The frequency with which the homeless were driven off by government staff before and during the pandemic is the core independent variable. The frequencies were divided into four categories in the following order: never, less frequently, as usual, and more frequently, labeled 1, 2, 3, and 4, respectively. Based on the demographic characteristics captured from participants and the previous literature on the homeless population, we selected nine control variables (see Table 4). This paper considered the self-reported health status and the demographic characteristics such as gender, place of birth, age, and education. Family responsibility and support, such as whether the participant had a child, whether the parents of the participant were alive, and whether the participant had brothers or sisters, how long they had been homeless were also included. There was a strong collinearity between the variable of marital status and whether the participant had a child. As a result, we did not include marital status and instead selected the variable of whether the participant had a child, which best represented the family responsibility. In addition, the relationships of the participant with family and the frequency of contact with family were also not included in the regression models because there were too much missing data (participants preferred not to say). The values assigned to the control variables are listed in Table 1.

#### Qualitative Data Analysis

Qualitative data were transcribed and analyzed by using an inductive approach (94, 95). The researcher read through the materials and then created a list of themes that emerged from the data (67). And then the researcher coded the data into the themes and selected quotes that represented the data coded into each theme (67). The key themes and subthemes we developed from the qualitative data are presented in Figure 1.

## Results

### Quantitative Findings

#### Basic Characteristics

In total, 103 eligible participants who experienced absolute homelessness were recruited. The geographical characteristics of respondents are shown in Figure 2, in which the absolute majority of the homeless lived in a densely populated city center where they could find enough food to survive even without any income. The basic characteristics of the samples are presented in Table 1. The age of participants showed a nearly normal distribution, with the majority aged 40–64 years. Of the 103 respondents to the survey, the majority were males (92.23%), while females accounted for only 7.77%; 29.13% were native Guangdong Province residents; and 70.87% came from outside Guangdong, attracted by the warm weather in Guangzhou City. The majority of participants (75.73%) had a low level of education (junior high school and below), and only two of the participants were college and university undergraduates; 66.99% were single (divorced-single and never married); 55.34% had no child at the time of the survey; and 60.19% of parents of the participants had already passed away, which means they had lost the most important family bond. Although 74.76% of participants had siblings, only 14.56% of them maintained a constant contact with family members, and only 25.24% reported that they had good relationships with family; 51.46% of participants had been homeless for more than 5 years; and 22 (21.36%) participants reported being homeless for <1 year, which indicates economic depression of China in the most recent year. Most importantly, among all participants, almost half (47.57%) reported feeling unhealthy. Many of the health problems or physical signs reported by participants, including experiencing a stroke (5); heart disease (4); hypertension (4); lung disease, such as emphysema, asthma, and bronchitis (4); diabetes mellitus (2); kidney disease (1); tumor (1); malnutrition and low immunity (1) were confirmed to be closely related to COVID-19 disease severity and fatality (96, 97). This suggests that the homeless are among the most vulnerable groups to COVID-19.

#### The Changes in the Lives of Homeless People Before and During the COVID-19 Pandemic

**Figure 3:**
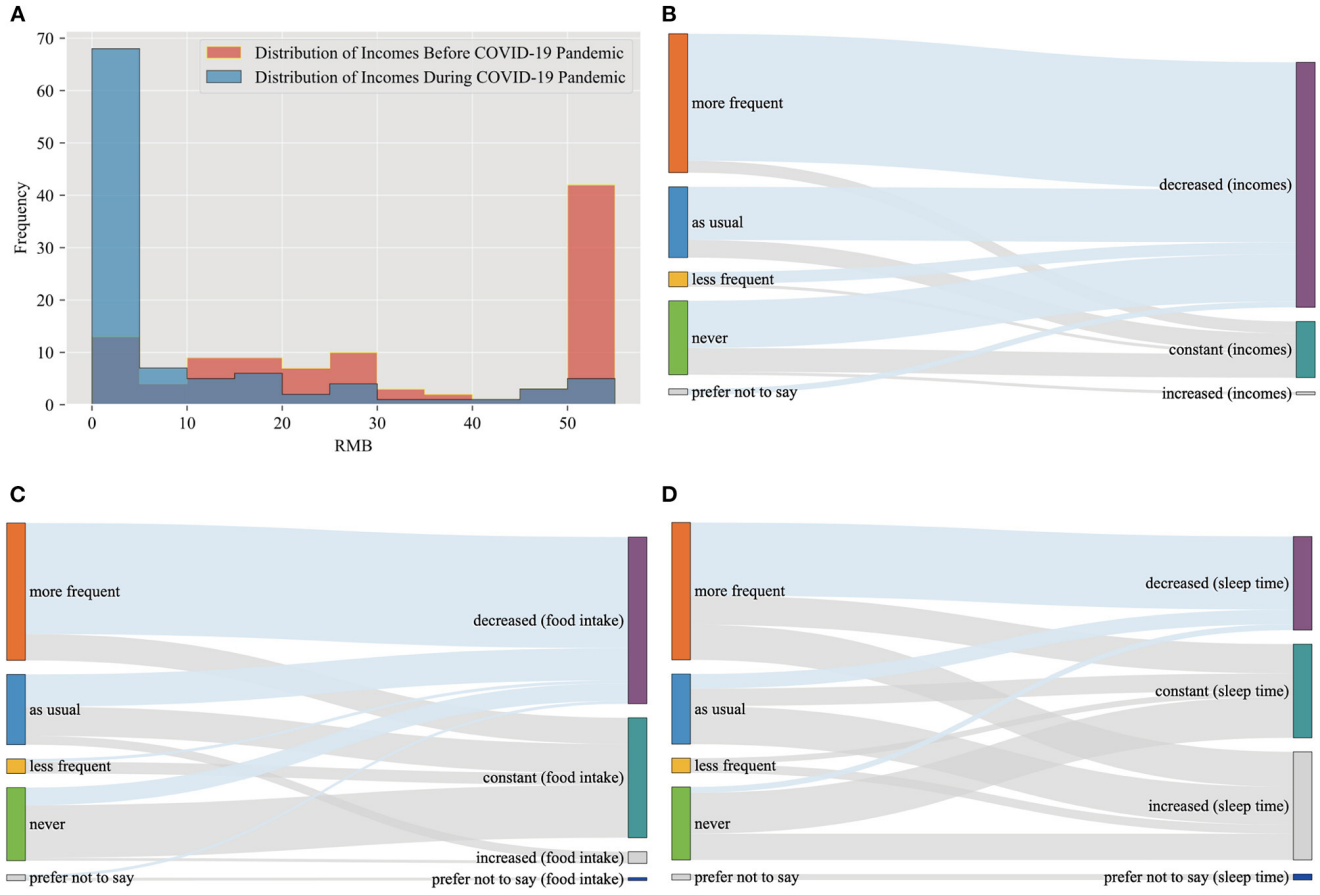
**(A)** The distribution of incomes before and during the COVID-19 pandemic; **(B)** the relationships between income changes and the frequency of being driven off; **(C)** the relationships between food intake changes and the frequency of being driven off; **(D)** the relationships between sleep time changes and the frequency of being driven off.

This paper attempted to reveal the changes in the lives of homeless people from three perspectives: income, food intake, and sleep time. Among the changes, we paid special attention to income because it forms the basis of daily life (98). Table 2 shows that collecting scraps to sell was the major income source (53.40%) for the homeless. However, the COVID-19 pandemic severely affected the incomes of the homeless. Few scraps can be found and sold for money from trash bins due to limited commercial activity. Moreover, local governments in China, such as Sichuan and Guangzhou, introduced comprehensive bans on rummaging through trash. Homeless individuals who were found searching through trash bins and containers were promptly stopped and driven away (38, 39). The implementation of this policy has caused the homeless to lose their main source of income. By comparing their incomes before and during the pandemic (see Table 2 and Figure 3A), we found that total income for homeless individuals fell sharply, while the number of homeless people who reported no income increased dramatically (from only 7.77% before the pandemic to 58.25% during the pandemic). The number of respondents who had relatively high-level incomes decreased significantly (>50 yuan, from 40.78% before the pandemic to only 4.85% during the pandemic). Overall, 80.58% of the participants reported a decline in income. Although 55.34% of participants reported a decrease in food intake, the impact of the pandemic on the food intake of the homeless seems to have lessened due to money-saving culture of China. Interestingly, the pandemic appears to have affected the sleep time of homeless people in different ways, with 31.07% of participants reporting a decline in sleep time, 31.07% reporting no change, and 35.92% reporting an increase in sleep time. The reason may lie on the scattering of homeless people and the uncertainty of encountering evictions from the government.

**Table 2:** The changes in the lives of homeless people before and during the pandemic (*n* = 103).

**Question**	**Frequency**	**Percentage (%)**
**Source of income**		
Collect scraps to sell	55	53.40
Charity from others	27	26.21
Other	23	22.33
Odd jobs	15	14.56
Government aid	10	9.71
Prefer not to say	3	2.91
**Income before the pandemic**		
No income (0 yuan)	8	7.77
0–5 yuan (not include 0 yuan)	5	4.85
5–10 yuan	4	3.88
10–15 yuan	9	8.74
15–20 yuan	9	8.74
20–25 yuan	7	6.80
25–30 yuan	10	9.71
30–35 yuan	3	2.91
35–40 yuan	2	1.94
40–45 yuan	1	0.97
45–50 yuan	3	2.91
>50 yuan	42	40.78
**Income during the pandemic**		
No income (0 yuan)	60	58.25
0–5 yuan (not include 0 yuan)	8	7.77
5–10 yuan	7	6.80
10–15 yuan	5	4.85
15–20 yuan	6	5.83
20–25 yuan	2	1.94
25–30 yuan	4	3.88
30–35 yuan	1	0.97
35–40 yuan	1	0.97
40–45 yuan	1	0.97
45–50 yuan	3	2.91
>50 yuan	5	4.85
**Income change**		
Decreased = 1	83	80.58
Constant = 2	19	18.45
Increased = 3	1	0.97
**Food intake change**		
Decreased = 1	57	55.34
Constant = 2	41	39.81
Increased = 3	4	3.88
Prefer not to say = None	1	0.97
**Sleep time change**		
Decreased = 1	32	31.07
Constant = 2	32	31.07
Increased = 3	37	35.92
Prefer not to say = None	2	1.94

#### Actions of Chinese Local Government Toward the Homeless During the COVID-19 Pandemic

Local Chinese government actions toward the homeless population are at odds because they include inhumane eviction measures as well as humanitarian aid such as sending homeless back to their hometowns and providing the homeless temporary shelters. This study focused on the inhumane efforts of the government by asking participants to compare the frequency with which they were driven off by government staff before and during the COVID-19 pandemic. As shown in Table 3, nearly half (45.63%) of the respondents reported that they were driven off more frequently during the pandemic, 23.3% reported they were driven off as usual, and only 4.85% of the participants reported being driven off less frequently, which confirmed that the local governments in China indeed considered driving away the homeless an important pandemic control measure. Interestingly, 24.27% of the participants had never encountered such a problem due to the mobility of the homeless and the irregularity of harassment from authorities. Consistent with our suggestion in the introduction that the humanitarian aid of the government might decrease, the proportion of the homeless who reported that the government had helped them during the pandemic (26.21%) decreased further compared to the already low proportion who reported receiving assistance before the pandemic (31.07%). In fact, based on the experiences shared with us by the homeless, it was mainly the NGOs, the temples, and even foreigners who had offered aid rather than the local governments. Overall, during the COVID-19 pandemic, humanitarian aid from governments decreased, whereas inhumane efforts to drive the homeless away intensified. Thus, there is no doubt that only 17.48% of the participants were satisfied with the performance of the government in supporting them during the pandemic (rated 4 and 5). Moreover, due to distrust toward the government, the majority of the homeless (74.76%) were unwilling to seek government assistance even during the pandemic.

**Table 3:** Action of the government toward the homeless before and during the pandemic (*n* = 103).

**Question**	**Frequency**	**Percentage (%)**
**Frequency of being driven off (compare before and during pandemic)**		
More frequently = 4	47	45.63
As usual = 3	24	23.30
Less frequently = 2	5	4.85
Never = 1	25	24.27
Prefer not to say = None	2	1.94
**Government initiatives to aid the homeless before the pandemic**		
No	71	68.93
Yes	32	31.07
**Government initiatives to aid the homeless during the pandemic**		
No	74	71.84
Yes	27	26.21
Prefer not to say	2	1.94
**Efforts of homeless people to seek government aid before the pandemic**		
No	78	75.73
Yes	23	22.33
Prefer not to say	2	1.94
**Efforts of homeless people to seek government aid during the pandemic**		
No	77	74.76
Yes	24	23.30
Prefer not to say	2	1.94
**Rate the performance of the government in supporting the homeless during the pandemic**		
1 = very poor	28	27.18
2 = poor	16	15.53
3 = fair	27	26.21
4 = good	15	14.56
5 = very good	3	2.91
Prefer not to say	14	13.59

*Homeless people rated the performance of the government on a Likert scale from 1 = very poor to 5 = very good with higher scores representing better performance*.

#### The Impact of the Inhumane Efforts of Government to Drive the Homeless Away on the Lives of the Homeless

To better understand the relationship between the inhumane efforts of the government to drive the homeless away and the lives of homeless people, we plotted Sankey diagrams (see Figures 3B–D). These diagrams visualize the flow of data to illustrate causality. By observing the relationships between the frequency with which the homeless were driven away and the income changes of homeless people (see Figure 3B), we noticed that the participants who were driven off more frequently during the pandemic had the highest proportion of decreasing income, while those who had never encountered such problems faced the lowest proportion of decreasing income. The higher the frequency of being driven away, the higher the proportion of declining income. As the frequency of being driven away increased, we observed a similarly higher proportion of a decreasing trend in food intake (see Figure 3C) and sleep time (see Figure 3D). The results of the ordered logit regression models further confirmed that the inhumane behavior of the government had a significantly negative impact on the lives of the homeless (see Table 4). The frequency of being driven off always had odds ratios lower than 1 and was always statistically significant in all our estimated models (with and without control variables), and the LR chi-square statistics associated with low *p*-values (at least <5%) indicated the good model fits. This indicates that with increasing frequency in driving the homeless away, the living standards of the homeless significantly declined. The Sankey diagrams and the results of the ordered logit regression models strongly confirmed our hypothesis and demonstrated the devastating impact of the strict pandemic control strategy of the government, which preferred to take inhumane actions, such as driving the homeless away, rather than providing more humanitarian aid to improve the lives of the homeless. The decline in living standards may further cause health problems in the homeless population.

**Table 4:** The ordered logit regression results estimating the effect of inhumane efforts of the government to drive the homeless away on the lives of homeless people (income, food intake, sleep time).

	**(1) Income change**	**(2) Income change**	**(3) Food intake change**	**(4) Food intake change**	**(5) Sleep time change**	**(6) Sleep time change**
	**Odds ratio**	**Odds ratio**	**Odds ratio**	**Odds ratio**	**odds ratio**	**odds ratio**
Frequency of being driven off	0.58** (−2.69)	0.45** (−2.56)	0.45** (−4.35)	0.43** (−3.59)	0.66** (−2.72)	0.60** (−2.68)
Gender		0.94 (−0.05)		1.79 (0.59)		3.67 (1.43)
Place of birth		1.61 (0.62)		0.61 (−0.81)		0.44 (−1.61)
Age		1.68* (2.49)		1.22 (1.43)		1.01 (0.05)
Education		0.71 (−0.44)		0.78 (−0.43)		0.76 (−0.54)
Self-reported health		1.91 (0.88)		1.74 (1.02)		1.13 (0.27)
Whether the participant had a child		0.45 (−0.92)		0.16** (−2.65)		1.16 (0.27)
Whether the parents of the participant were alive		2.25 (0.94)		1.25 (0.34)		1.36 (0.55)
Whether the participant had brothers or sisters		5.74 (1.50)		0.74 (−0.43)		0.71 (−0.54)
How long the participant had been homeless		1.03 (0.19)		0.90 (−1.09)		1.19 (1.94)
Number of observations	101	84	101	84	101	84
LR chi-Square	7.46**	21.66*	21.98**	29.56**	7.73**	18.31*

“**” and “*” indicate significance levels of 1 and 5%, respectively. z-statistics are presented in parentheses.

Notably, age had an odds ratio higher than 1 in Model (2) with statistical significance, which means that older homeless people with considerable homeless experience might be more able to survive during this difficult time. It is particularly noteworthy that the homeless people who had a child faced a significant decline in food intake (odds ratio lower than 1 with statistical significance), and, thus, need urgent and special attention.

### Qualitative Findings

The qualitative data are presented below, using headings to describe each key theme and subtheme (see Figure 1) (91). The themes are illustrated by using examples where appropriate.

#### Actions of Local Government Toward the Homeless

As discussed in the introduction section, the local government of China manages the homeless in cities in three ways: sending them back to their hometowns, providing them with temporary shelters, and driving them off. The first two actions seem humane, but the last one belongs to the inhumane measures.

##### Humanitarian Aids

Consistent with our previous analysis, local governments stopped sending the homeless back to their hometowns due to the strict lockdown measures during the COVID-19 pandemic.

“*In March, I went to the rescue station and asked them to help me buy a ticket home. However, they refused and blamed it on the pandemic.” (Respondent: 030)*.

Meanwhile, the local rescue stations (homeless shelters in China) lacked the will and the ability to help the homeless during the pandemic.

“*The doors of the rescue stations were tightly closed during the pandemic. They didn't take in the homeless people.” (Respondent: 016)*

“*Help us? Help my ass! They simply did not come. Last time there was an old man, more than 80 years old, almost dying. We called the rescue station for help, while the rescue station said: ‘we cannot help, go to the police station!’ So, we went to the police station, and then the police and the ambulance came and took the old man to the hospital… The rescue station just stopped helping homeless people.” (Respondent: 040)*

“*When I really needed help, no one came to help me. When I didn't need help (around March or April when the pandemic had been effectively contained and all the commercial facilities had been reopened), they came out to help us. I even yelled at them for that.” (Respondent: 067)*

Interestingly, it was mainly the NGOs, the Buddhists, and even foreigners who had offered aid rather than the local governments.

“*The government did not come to help us, but the foreigners came three times a week, handing out some noodles and masks” (Respondent: 048)*

“*During the pandemic, we ate one meal a week. Sometimes young volunteers would bring food, Buddhists would give us cookies and rice, and the foreigners would also bring us some food. We survived on these food for 90 days, otherwise, a lot of people would have starved to death!” (Respondent: 103)*

However, even in the rescue station, homeless people were treated without respect, which also makes most homeless reluctant to seek government assistance even during the pandemic.

“*I went to a rescue station, but people there insisted on taking my phone away. I got angry and said, ‘I came to receive help, not to have my freedom restricted.’ I quarreled with them and left.” (Respondent: 064)*.

“*Oh, it was disgusting! Pigs were eating better food than us. The food at the rescue station was boiled cabbage, but it was too hard to be edible. They bought pork skins that stank. The seller had sold off the edible ones and left the lousy ones there, piles after piles that had been unmoved for two weeks. No one cleaned them, and they were cooked for us to eat. The staff at the rescue station were devils. …The government never treated us like human beings.” (Respondent: 099)*.

##### Inhumane Eviction Efforts

The humanitarian aid provided by the government was reduced, while inhumane efforts to drive the homeless away intensified.

“*The eviction measures are stricter during the pandemic… They always came to drive us away.… They came to check on us every day.” (Respondent: 007)*

“*We were being driven off because they feared that we were infected. But we were not, we are just part of the lowest social hierarchy.” (Respondent: 015)*

“*During the pandemic, the urban administration, in conjunction with the police station, drove us away and did not even allow us to find a place to sit” (Respondent: 016)*

“*The urban management officers claimed that ‘crossing the road and you'd enter Liuhua, which is not part of our jurisdiction.’ However, Liuhua officials forced us to return. Can you name another government that would act like this? A government should solve people's problems, right? Instead, they were shifting the responsibilities like kicking a football between two sides.” (Respondent: 040)*

“*He ordered me to move to another district and said you can't live here. I said, ‘I have been living here for decades, and I am not familiar with other districts. What do you want me to do there?’” (Respondent 090)*.

#### The Impact of the Inhumane Efforts of the Government

##### Income

During the COVID-19 pandemic, incomes decreased dramatically due to the strict pandemic control strategy which preferred to take inhumane eviction actions, rather than providing more humanitarian aid.

“*The urban management officer kept driving us away and we couldn't pick up the garbage.” (Respondent: 015)*

“*I am living on the ground stall… However, no stalls were allowed during the pandemic, so I can only live by collecting garbage.” (Respondent: 065)*

“*During the pandemic, a gathering was strictly forbidden, so we could not pick up anything to sell and had no income. We could only pick up some leftover food and rotten fruits to satisfy our hunger.” (Respondent: 068)*

##### Food

The decrease in income was followed by a shortage of food. The inhumane measures of the government also negatively impact the food intake of the homeless.

“*I didn't make any money during the pandemic, so I had to live on steamed buns and pickles (the cheapest food in China).” (Respondent: 046)*

“*During the pandemic, I could only go to the trash can at night to pick up leftover food to eat. Because if the urban management officer caught us picking up food from the trash, they would drive us away.” (Respondent: 021)*

“*At that time, there was nothing to eat in the garbage cans, because people in the community were restricted from going out and could not come out to consume, and the garbage in the community was not allowed to be thrown outside directly.” (Respondent: 068)*

Meanwhile, the humanitarian aid of the government for homeless people also decreased significantly, aggravating the difficulty in getting enough food.

“*The rescue station had crackers and water last year, but none this year. When the rescue station's vehicle arrived, I asked if they could provide us with crackers today. He (the staff member from the rescue station) said no. He shook his head, entered the vehicle, and did not get out again. He did not care about our situation.” (Respondent: 040)*

“*The rescue station is packed with people. He did not rescue anyone but handed a pack of instant noodles to me and said, ‘Bug off.’ I did not go in, and I took two packs of instant noodles to eat.” (Respondent: 068)*.

“*No one ever offered any help to us during the outbreak. The government feared that the virus may hit the rescue station, so it only gave two packs of instant noodles to whoever came. That is to say, I had to take two packs of Huafeng instant noodles and leave. Moreover, they took your photograph before you took the instant noodles. I stopped going there.” (Respondents: 092)*

Moreover, food aid from NGOs was strictly forbidden under aggressive pandemic prevention and control.

“*They (government staff) drove the volunteers (from NGOs) away and threw the rice and bread on the ground. What other countries would do that? However, we can only stay silent.” (Respondent: 015)*

“*They did not allow the volunteers to distribute food, we could not get the food.” (Respondent: 065)*.

“*The volunteers had to distribute food surreptitiously because it was not allowed. The governments were scared about potential cross-infection from the gathering of homeless people.” (Respondent: 064)*.

##### Sleep

Lots of homeless people reported that they were constantly being driven from place to place by governments during the pandemic, resulting in a decline in sleep time.

“*When the COVID-19 outbreak was at its height, they had three inspections a day: 8:30–9:00, 12:00–13:00, and 21:00–22:00. You could not go to bed until they completed the inspections.” (Respondent: 028)*

“*During the pandemic, we were chased around by the urban management officers, and I could not sleep at night. No matter where you slept, the urban management officers came and chased you…How do you find time to sleep? The urban management officers came four or five times a day, so I couldn't sleep during the pandemic.” (Respondent: 042)*

“*When the WHO officials arrived, regulations were tight. Many people who had been sleeping in bridge openings were driven away. The officials called on them to sleep in inconspicuous places (like an alley entrance) to avoid being spotted.” (Respondent: 068)*

“*During the COVID-19 pandemic, I had to go to bed at 1:00 am because the urban management officers came to the place at 12:40 and rinsed where we slept with water.” (Respondent: 104)*

##### Mental Health and Attitude Toward Government

Homeless people are particularly vulnerable to the impact of the COVID-19 crisis. Under the strict pandemic control strategy of the government, they had a particularly difficult time and suffered a lot of stress, anxiety, and depression.

“*During the Chinese New Year, I was scared to death, and there was no food to eat…I was so sad at that time. Even now when I think about it, I am feeling weepy.” (Respondent: 040)*

“*I didn't dare to think about it. In retrospect, it was horrible, and I had no idea how I managed to get through it. I could only afford a piece of bread daily. Five yuan for one loaf, so I'd be anxious if I had it all for one serving.… During the pandemic, everyone was fearful of us because they thought we were spreading the virus, so they kept their distance from us.… I was so lonely that I even considered committing suicide. I'd see several people in the daytime, but I'd be completely alone at night.” (Respondent: 067)*

“*I was very tired, very tired of living, and very depressed.” (Respondent: 073)*

The lack of humanitarian assistance and continued expulsion and discrimination also sowed the seeds of distrust and even hatred toward the government and society in the hearts of the homeless population.

“*I don't believe them. They're all liars.” (Respondent: 003)*

“*To be honest, I would have killed an urban management officer if they had bothered me.” (Respondent: 067)*

“*The government doesn't care about your situation. They gave up on the underprivileged. When the government gives up on you, you have to—no matter if you admit it or not—‘fend for yourself’. When the government cannot save enterprises, how can you count on it to save the homeless? The government would only save big enterprises.” (Respondent: 068)*.

“*No one cared about our situation, people just want us to die as soon as possible. This is true because China has a large population. Some regard us as a burden on society. We are treated like garbage, are we not?' (Respondent: 099)*.

## Discussion

To the best of our knowledge, this is the first survey-based study to present the characteristics, particular the health status, of the street homeless group in mainland China, and the first article to explore their living situation during the COVID-19 pandemic. This is also the first empirical study to investigate the effect of the strict pandemic control strategy of Chinese government on the lives of the homeless population.

There are four main findings of this study. First, the homeless people in Guangzhou tended to be male, aged 40 to 64 years, less well-educated, originally from outside Guangdong Province, and living in a densely populated city center. Most homeless people have no family responsibilities or family support. Most were single with no children, and most of their parents were no longer alive. Although they might have brothers or sisters, they barely had any contact with family members, and most had poor relationships with their families. After experiencing homelessness for some time (more than half of the participants had been homeless for more than 5 years), almost half of the homeless had poor health with medical conditions, such as experiencing a stroke, heart disease, and hypertension, which made them extremely vulnerable to COVID-19. Second, the pandemic caused a huge decline in incomes of homeless people while having less of a declining impact on food intake due to money-saving culture of China. Interestingly, the pandemic affected sleep time of homeless people in different ways due to their scattered locations and the uncertainty of encountering evictions. Third, during the COVID-19 pandemic, humanitarian aid from the local governments of China decreased, whereas inhumane efforts to drive the homeless away increased, which was also confirmed by our qualitative data. Nearly half of the respondents reported that they were driven off more frequently during the pandemic, indicating that local governments in China indeed used driving away the homeless as a key pandemic control strategy. Finally, Sankey diagrams, the results of the ordered logit regression models, and qualitative materials strongly confirmed our hypothesis and demonstrated the devastating impact of the strict pandemic control strategy of the government on the homeless. The strategy preferred inhumane efforts, such as driving the homeless away rather than providing more humanitarian aid to improve the lives of the homeless.

Our qualitative materials also revealed some other important conclusions. First, the strict pandemic control of the government had a negative impact on the mental health of the homeless and their trust in the government. Relief agencies lack effective policy formulation and implementation procedures during the control period. Second, NGOs and Buddhist believers played an important role in the rescue of the homeless. Social organizations have become substitutes and the main supplements of government in the rescue procedures for vulnerable groups. Third, other government departments, for example, the police station, provided emergency assistance to the homeless, which means the linkage mechanism of various government departments does not exist in the rescue, and the unclear responsibilities made the process of seeking assistance aggravated. In addition, the quantitative models also revealed that the homeless people who had child faced a significant decline in food intake and, thus, need urgent and special attention.

The homeless in China have long suffered abusive treatment, and it is time to make a change. Although the Chinese government has made a remarkable progress in supporting the homeless population since 2003, it is far from enough. The homeless population in China, which is among the most vulnerable groups to the COVID-19 pandemic, should not be forgotten. Everyone, including the homeless, should be treated with dignity and respect. In the field of social assistance, government departments may face a situation that lacks policy formulation and implementation. They should consider the functions of social organizations and non-profit organizations, free up space for their participation, and give policy support in the assistance to vulnerable groups. With the cold weather approaching, more temporary shelters with food, safe water, toilets, heating devices, sanitation, and hygiene supplies are urgently required. Additional separate spaces with adequate medical infrastructure to isolate and care for the homeless who are diagnosed with COVID-19 urgently need to be established after COVID-19. In addition, the mental health of the homeless should be addressed and accessible psychosocial support services should be provided.

This study had some limitations. Although we tried our best to find eligible participants through various means, some street homeless may have been overlooked by our research teams and not included in our survey, which may cause a potential selection bias. Females may be underrepresented in this study; therefore, findings related to gender differences should be considered preliminary and interpreted with caution.

## Conclusions

This paper is the first to explore the characteristics of the street homeless population in mainland China and their living situation during the COVID-19 pandemic. Most importantly, this study demonstrates the devastating effect of the strict pandemic control of the Chinese government, which was considered a great success (99, 100), on the lives of homeless people. Pandemic control measures are vital, but homeless people, who are particularly vulnerable to the COVID-19 pandemic, should not be sacrificed. Urgent measures such as providing more temporary shelters and addressing the mental health issue should be taken to ensure the protection of the homeless population and prevent any impending humanitarian crisis.

## Data Availability Statement

The raw data supporting the conclusions of this article will be made available by the authors, without undue reservation.

## Ethics Statement

The studies involving human participants were reviewed and approved by School of Public Administration, Nanfang College of Sun Yat-sen University. Written informed consent for participation was not required for this study in accordance with the national legislation and the institutional requirements.

## Author Contributions

LH conceived of the study, conducted the survey, collected and analyzed the data, and prepared the manuscript draft. YW provided comprehensive editing, interpretation of the data, and refinement of the manuscript. SZ, TD, YC, WC, CW, YZ, and HZ conducted the survey and collected the data. All authors read and approved the final manuscript.

## Conflict of Interest

The authors declare that the research was conducted in the absence of any commercial or financial relationships that could be construed as a potential conflict of interest.

## Publisher's Note

All claims expressed in this article are solely those of the authors and do not necessarily represent those of their affiliated organizations, or those of the publisher, the editors and the reviewers. Any product that may be evaluated in this article, or claim that may be made by its manufacturer, is not guaranteed or endorsed by the publisher.
